# Supplementary material for: Hai||om children mistrust, but do not deceive, peers with opposing self-interests
Source: PLoS One. 2020 Mar 10;15(3):e0230078. doi: 10.1371/journal.pone.0230078 (PMC7064192; doi:10.1371/journal.pone.0230078)
Supplement: S1 Fig — E = Experimenter; S = Sender; R = Receiver; (a) S and R observe E putting the balls on the plates; (b) R turns around while E shows S the location of the reward (here: candy is on plate closer to E); (c) S places the stick on one plate to indicate the location of the reward (Deception assessed); (d) R chooses while S turns away (Mistrust assessed); (e) balls are stored according to R’s choice before S turns back to the scene; hypothesis-conform behaviors are illustrated with honesty and trust during cooperation and deception and mistrust during competition. (DOCX) [file pone.0230078.s001.docx]

S1 Fig
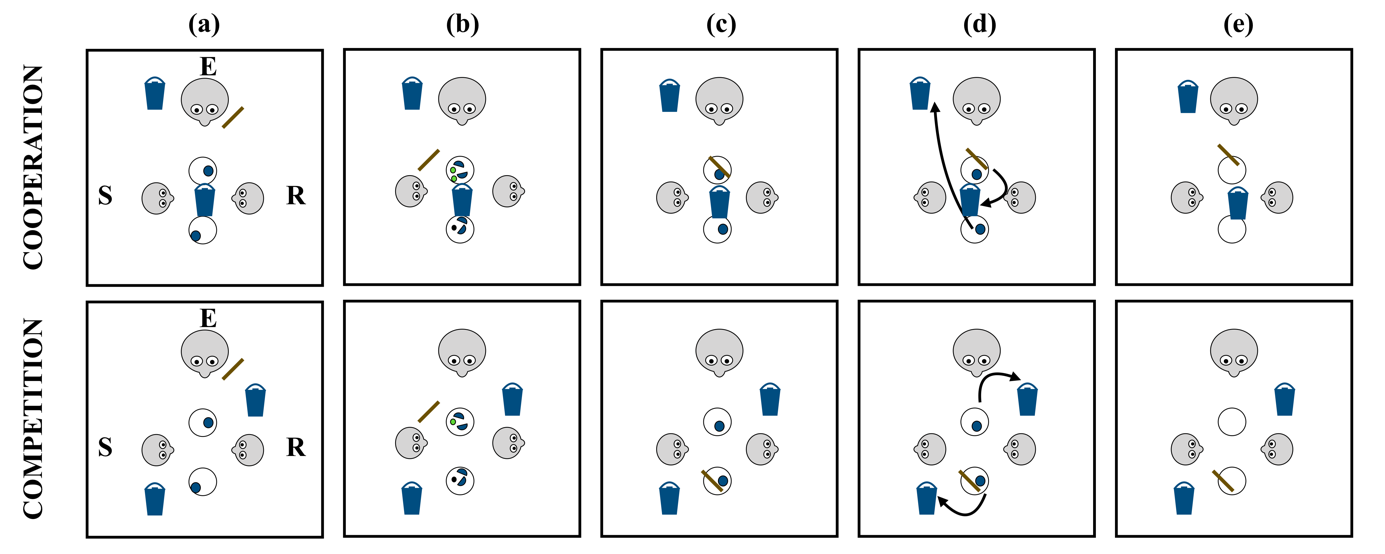


*S1 Fig*: Schematic procedure in both conditions. E = Experimenter; S = Sender; R = Receiver; (a) S and R observe E putting the balls on the plates; (b) R turns around while E shows S the location of the reward (here: candy is on plate closer to E); (c) S places the stick on one plate to indicate the location of the reward (Deception assessed); (d) R chooses while S turns away (Mistrust assessed); (e) balls are stored according to R’s choice before S turns back to the scene; hypothesis-conform behaviors are illustrated with honesty and trust during cooperation and deception and mistrust during competition
