# Supplementary material for: Hai||om children mistrust, but do not deceive, peers with opposing self-interests
Source: PLoS One. 2020 Mar 10;15(3):e0230078. doi: 10.1371/journal.pone.0230078 (PMC7064192; doi:10.1371/journal.pone.0230078)
Supplement: S1 Table — (a) Hypothesis 1; (b) Hypothesis 2; (c) Hypothesis 3; Numbers in cells reflect number of children showing the respective behaviors; DEC = Sender deceives; HON = Senders gives honest hint; MIS = Receiver mistrusts the sender; TRU = Receiver trusts the sender; each child engaged in each role (sender & receiver) in two trials throughout the study and without receiving feedback between trials. (DOCX) [file pone.0230078.s002.docx]

S1 Table

|  | **(a)**  **H1: Deception as Sender** | |  | **(b)**  **H2: Mistrust as Receiver** | |  |  | **(c)**  **H3: Deception**  **~ Mistrust** | |
| --- | --- | --- | --- | --- | --- | --- | --- | --- | --- |
|  | **DEC**  **1** | **HON**  **0** |  | **MIS**  **1** | **TRU**  **0** |  |  | **MIS**  **1** | **TRU**  **0** |
| Competition | 15 | 49 |  | 34 | 30 |  | DEC  1 | 15 | 38 |
| Cooperation | 10 | 54 |  | 19 | 45 |  | HON  0 | 10 | 65 |

*S1 Table:* Raw data. (a) Hypothesis 1; (b) Hypothesis 2; (c) Hypothesis 3; Numbers in cells reflect number of children showing the respective behaviors; DEC = Sender deceives; HON = Senders gives honest hint; MIS = Receiver mistrusts the sender; TRU = Receiver trusts the sender; each child engaged in each role (sender & receiver) in two trials throughout the study and without receiving feedback between trials
