# Supplementary material for: Hai||om children mistrust, but do not deceive, peers with opposing self-interests
Source: PLoS One. 2020 Mar 10;15(3):e0230078. doi: 10.1371/journal.pone.0230078 (PMC7064192; doi:10.1371/journal.pone.0230078)
Supplement: S2 Table — (DOCX) [file pone.0230078.s003.docx]

S2 Table

| **Fixed Effects** | ***H1: Deception as Sender*** | | |  | ***H2: Mistrust as Receiver*** | | |
| --- | --- | --- | --- | --- | --- | --- | --- |
|  | **Estimate** | **SE** | ***p*** |  | **Estimate** | **SE** | ***p*** |
| ***Predictors*** |  |  |  |  |  |  |  |
| (Intercept) | 0.848 | 1.602 | .597 |  | -0.552 | 1.472 | .708 |
| Age | -0.286 | 0.283 | .231 |  | 0.148 | 0.216 | .493 |
| Condition | -0.482 | 0.473 | .309 |  | -1.077 | 0.424 | .011* |
| ***Controls*** |  |  |  |  |  |  |  |
| Sex | -0.342 | 0.487 | .483 |  | -0.152 | 0.422 | .719 |
| Trial | 0.104 | 0.456 | .820 |  | -0.510 | 0.388 | .189 |

*S2 Table:* Model outputs for hypotheses 1 and 2, **p* < .05
