# Supplementary material for: Hai||om children mistrust, but do not deceive, peers with opposing self-interests
Source: PLoS One. 2020 Mar 10;15(3):e0230078. doi: 10.1371/journal.pone.0230078 (PMC7064192; doi:10.1371/journal.pone.0230078)
Supplement: S3 Table — (DOCX) [file pone.0230078.s004.docx]

S3 Table

| **Fixed Effects** | ***Vigilance in either role*** | | |
| --- | --- | --- | --- |
|  | **Estimate** | **SE** | ***p*** |
| ***Predictors*** |  |  |  |
| (Intercept) | -0.003 | 1.929 | .999 |
| Age | -0.124 | 0.277 | .654 |

*S3 Table:* Additional model to test whether correlation between mistrust and deception varies by age (Outcome/Vigilance: 1 = child deceives AND mistrusts; 0 = remaining children)
