# Supplementary material for: Hai||om children mistrust, but do not deceive, peers with opposing self-interests
Source: PLoS One. 2020 Mar 10;15(3):e0230078. doi: 10.1371/journal.pone.0230078 (PMC7064192; doi:10.1371/journal.pone.0230078)
Supplement: S1 File — (DOCX) [file pone.0230078.s005.docx]

**Supplementary Material II**

**– Codebook –**

POPULATION: [Haikom]

Cultural context in which data was assessed

DYAD: [1 - 32 ]

Running number of dyads

ID: [e.g., 01_Coop_F_1] – e.g., dyad 1_cooperation_female_child 1

ID-variable for each participant, displays counterbalancing for condition, sex, and location of rewards

SEX: [F; M] – Female, Male

Sex of participant

AGE: [4.51 - 8.82]

Age of participant in decimal numbers

CONDITION: [COOP; COMP] – Cooperation; Competition

Condition in which dyad was assessed

TRAINING1: [0 - 4]

Number of trials in which participant won candy during Guessing Game I

MISTRUST1: [0; 1] – Trust; Mistrust

Participant’s behavior in her first trial as a receiver

DECEPTION1: [0; 1] – Honesty; Deception

Participant’s behavior in her first trial as a sender

MISTRUST2: [0; 1] – Trust; Mistrust

Participant’s behavior in her second trial as a receiver

DECEPTION2: [0; 1] – Honesty; Deception

Participant’s behavior in her second trial as a sender
