# Supplementary material for: Hai||om children mistrust, but do not deceive, peers with opposing self-interests
Source: PLoS One. 2020 Mar 10;15(3):e0230078. doi: 10.1371/journal.pone.0230078 (PMC7064192; doi:10.1371/journal.pone.0230078)
Supplement: Attachment — Submitted filename: Response to Reviewers.docx [file pone.0230078.s007.docx]

Dear Professor Dr. Butler:

Thank you for the opportunity to revise our manuscript PONE-D-19-28205. We are grateful for the positive evaluation provided by yourself and the reviewers.

We have addressed the point raised by reviewer 2 in the manuscript.

**Reviewer 2**

**(1)**

**“One minor comment: I would ask that the authors append the analyses they performed in response to my comments (e.g. the practice trial analysis) into the actual manuscript or supplement itself so that it's accessible to readers.“**

*We now mention this analysis in the manuscript. We have added random intercepts of ID to these models to account for within-subject variance. The results remain unaffected by doing so.*

“The number of rewards children received during Guessing Game I was not included to the model because of convergence issues. In a separate analysis, this variable was not linked to children’s deception (χ^2^ (1) = 0.354, *p* = .552).“ (p. 11)

“The number of rewards children received during Guessing Game I did not predict their subsequent mistrust (χ^2^ (1) = 0.327, *p* = .568).“ (p. 11)
